# 

**Published:** 1873-02

**Authors:** 


TO OUR SUBSCRIBERS.—We thank our friends who have so promptly forwarded their subscriptions. They will find credits given them in this number. In future we will give in the journal the names of all paying, and amounts paid, which we design to have all the force of a receipt. All who have not remitted we hope will do so at once, as, by our terms with the publisher, the RECORD will be conducted strictly upon the *cash basis.* We trust all will remit their dues at once, with the promise on our part that we shall spare no effort to give them a first-class medical journal. All letters and communications in the future must be directed to Powell & Goldsmith, box 527, Atlanta, Georgia.

Communications.—The following communications have been received: "Chronic Diarrhœa as it occurred during and since the war," by Alban S. Payne, M.D., of Virginia; "Yellow Fever," by T. A. Cooke, M.D., of Louisiana.

List of Payments Received for the Record, Commencing with the January Number.—Dr. E. J. Palmer, Georgia, $2 50; Dr. J. A. Hanks, North Carolina, 2 50; Dr. Edward Mann, North Carolina, 2 50; Dr. A. J. Lutherloh, North Carolina, 2 50; Dr. J. H. Cook, North Carolina, 2 50; *Dr. J. W. Taylor, Georgia, 2 00; Dr. W. M. Fitch, South Carolina, 2 50; Dr. J. T. Hargroves, Alabama, 2 50; Dr. R. G. Harper, Georgia, 2 50; *Dr. C. C. Jones, Alabama, 2 00; Dr. R. S. Hallsey, North Carolina, 2 50; Dr. T. J. DuBose, South Carolina, 2 50; Dr. D. R. Fox, Louisiana, 2 50; Dr. W. W. Duffee, Georgia, 2 50; Dr. Robert Kells, Mississippi, 2 50; Dr. J. T. Sego, Georgia, 2 50; Dr. J. W. Gilbert, Alabama, 2 50; Dr. Thomas A. Cook, Louisiana, 2 50; Dr. T. P. Oliver, Georgia, 2 50; Dr. A. S. Payne, Virginia, 2 50; Dr. J. B. Manson, Georgia, 2 50; Dr. W. R. Bell, Georgia, 2 50; Dr. A. C. Randolph, Virginia 2 50; Dr. B. T. Rea, Alabama, 2 50; Dr. J. W.

Talley, Georgia, 2 50; Dr. W. P. Fears, Texas, 2 50; Dr. W. A. Moore, Georgia, 2 50; Dr. J. T. Dixon, Georgia, 2 50; Dr. J. W. Bennett, North Carolina, 2 50; Dr. T. F. Green, Georgia, 2 50; Dr. A. G. Whitehead, Georgia, 2 50; Dr. L. G. Attaway, Georgia, 2 50; Dr. W. H. Boxley, Georgia, 2 50; Dr. J. A. Perkins, Georgia, 2 50; *Dr. B. W. Taylor, Florida, 2 00; Dr. A. H. Randle, Georgia, 2 50; Dr. W. Offeet, Louisiana, 2 50; Dr. J. S. Baker, Georgia, 2 50; Dr. W. B. Williamson, Mississippi, 2 50; Dr. John Hockenhull, Georgia, 2 50; *Dr. J. W. Whitmore, Mississippi, 2 00; *Dr. C. A. W. Bostick, Georgia, 2 00; Dr. B. F. Chapman, Georgia, 2 50; Dr. G. H. Thompson, Alabama, 2 50; Dr. A. Rives, Mississippi, 2 50; *Dr. J. P. Cheney, Georgia, 2 00; Dr. A. T. Cheatham, Georgia, 2 50; Dr. John Elsmer, Colorado Territory, 2 50; *Dr. H. Kelly, North Carolina, 2 00; *Dr. M. W. Hill, North Carolina, 2 00; Dr. W. C. Musgrove, Georgia, 2 50; Dr. H. M. Talley, Georgia, 2 50; Dr. E. Y. Fleming, Mississippi, 2 50; Dr. F. B. Henderson, Alabama, 2 50; Dr. S. M. Hogan, Alabama, 2 50; Dr. W. L. Thomason, Alabama, 2 50; Dr. N. M. Bledsoe, Alabama, 2 50; Dr. Zeb. T. Murphy, Alabama, 2 50; Dr. Robert Powell, Virginia, 2 50; Dr. William R. Whitehead, Colorado Territory, 2 00; Drs. Lanier & McCabe, Mississippi, 2 50; Dr. N. J. Pittman, North Carolina, 2 50; *Dr. S. E. Winnemore, Alabama, 2 00; Dr. John H. Henry, Alabama, 2 50; Dr. W. D. Hoyt, Georgia, 2 50; Drs. Huggins & Prather, Mississippi, 2 50; Dr. O. P. Langworthy, Louisiana, 2 50; Dr. J. M. Williams, Mississippi, 1 00; Dr. J. C. Brown, Georgia, 2 50; Dr. E. A. Andison, North Carolina, 2 50; Dr. M. T. Anderson, Mississippi, 2 50; Dr. A. H. Smith, Mississippi, 2 50; Dr. George E. Redwood, Mississippi, 2 50; Dr. Edward M. Millard, Louisiana, 2 50; Dr. W. J. Hays, Alabama, 2 50; Dr. E. L. Calhoun, Georgia, 2 50; Dr. J. M. Toner, Washington, D.C., 2 50; Dr. Alex. J. Kolb, Louisiana, 2 50; Dr. W. H. Page, Georgia, 2 50; Dr. J. T. Foster, Arkansas, 2 50; Dr. J. W. Jones, Louisiana, 2 50; Dr. Thomas S. Jones, Louisiana, 2 50; Dr. A. A. Carruth, Louisiana, 2 50; Dr. J. J. Horton, South Carolina, 2 50; Dr. Thomas Nicholson, Louisiana, 2 50.

Those to whose names the * is affixed, not knowing, probably, that the subscription price has been placed at $2 50, sent only $2 00, fifty cents being still due. Those who have paid for last year, but who have not received receipts, will be credited as above in our next issue.

### The Southern Medical Record.

EDITED BY

### T. S. POWELL, M.D.,

AND

### W. T. GOLDSMITH, M.D.

---

### ASSOCIATE EDITORS:

**GEORGIA.**

Robert Battey, M.D.
R. C. [illegible]ord, M.D.
W. L. Kirkpatrick, M.D.
James A. Long, M.D.
W. L. M. Harris, M.D.
H. L Battle, M.D.
W. C. Musgrove, M D.
L B. Bouchelle, M.D.
John C. Drake, M.D.
E. F. Knott, M.D.
Thomas Burdell, M.D.
E. H. W. Hunter, M.D.
V. S. Hitt, M.D.
J. E. Pope, M.D.
J. A. Hunnicutt, M.D.
A. H. Brantley. M.D.
J. J. Knott, M.D.
J. C. Wisdom, M.D.
W. W. Leake, M.D.

**ALABAMA.**

J. C. Knox, M.D.
Geo. F. Taylor, M.D.
J. B. Barnett, M.D.
S. M. Hogan, M.D.
D. S. Hopping, M.D.
J. T. Searcy, M.D.
J. F. Hill, M.D.

**MISSISSIPPI.**

C. B. Gallaway, M.D.
Edward Lea, M.D.
S. V. D. Hill, M.D.
W. B. Harvey, M.D.
J. W. M. Shattuck, M.D.
E. T. Henry, M.D.
T. P. Lockwood, M.D.
Robert Kells, M.D.

**VIRGINIA.**

Charles S. Lewis, M.D.
John H. Claiborne, M.D.
F. Horner, Jr., M.D.
R. J. Preston, M.D.
A. S. Payne, M.D.
J. E. Lockridge, M.D.
J. S. Wharton, M.D.
Wm. O. Owen, M.D.
Sam'l P. Ripley, M.D.
Benj. Blackford, M.D.
E. A. Drewry, M.D.
Rob B. Tunstall, M.D.
A. J. Terrell, M.D.
W. F. Barr, M.D.
L. B. Anderson, M D.
S. L. Jepson, M.D., W. Va.
J. M. Lazzell, M.D.

**SOUTH CAROLINA.**

E. T. McSwain, M.D.
G. M. Rivers, M.D.

**TENNESSEE.**

J. D. Tucker, M.D.
Swan M. Burnett, M.D.

**TEXAS.**

S. M. Welch, M.D.
T. J. Heard, M.D.
W. A. Heard, M.D.

**ARKANSAS.**

A. D. Hodge, M.D.
J. K. Hodge, M.D.

**NORTH CAROLINA.**

J. R. Hughes, M.D.
S. S. Satchwell, M.D.
A. B. Pierce, M.D.
H. Kelly. M.D.
Geo. A. Foote, M.D.
E. A. Anderson, M.D.
H. T. Bahnson, M.D.
Willis Alston, M.D.
Thos. F. Wood, M.D.
N. J. Pittman, M.D.

### CORRESPONDING EDITORS:

J. M. Toner, M.D., Washington City, D. C.
John H. Weir, M.D., Illinois.
Thomas J Gallaher, M. D, Pennsylvania.
Ely Van DeWarker, M. D., New York.
Robert Robson, M D., Indiana.
C. C. F. Gay, M.D., New York,
*Surgeon of Buffalo General Hospital.*
W. T. Taylor, M.D , Kentucky.
J. Orne Green, Boston, Massachusets.
B. W. Stone, M.D., Kentucky,
*Assistant Physician Western Lunatic Asylum.*
James Tyson, M.D., Philadelphia, Pa.
W. W. Leen, M.D., Philadelphia.

M. H. Henry, M.D., New York,
*Surgeon to New York Dispensary, Department of Venerial and Skin Diseases.*
Wm. R. Whitehead, M.D., Colorado Territory.
A. Patton, M.D., Indiana.
Benjamin Howard, M.D., New York.
Chas Kearns, M.D., Kentucky.
S. C. Busey, M.D., Washington, D. C.
A. W. Hobson, M.D., Arkansas.
A. W. Calhoun, M.D., now of Berlin, Prussia.
T. Curtis Smith, Ohio.
Geo. Strawbridge, M.D., Philadelphia.

---

☞ *All Business Letters should be addressed to POWELL & GOLDSMITH, Post Office Box* 527, *Atlanta, Georgia.*

